# Gene expression profiling identifies inflammation and angiogenesis as distinguishing features of canine hemangiosarcoma

**DOI:** 10.1186/1471-2407-10-619

**Published:** 2010-11-09

**Authors:** Beth A Tamburini, Tzu L Phang, Susan P Fosmire, Milcah C Scott, Susan C Trapp, Megan M Duckett, Sally R Robinson, Jill E Slansky, Leslie C Sharkey, Gary R Cutter, John W Wojcieszyn, Donald Bellgrau, Robert M Gemmill, Lawrence E Hunter, Jaime F Modiano

**Affiliations:** 1 Integrated Department of Immunology, University of Colorado, Denver, School of Medicine (UCD SOM), Denver, CO, USA; 2 Department of Medicine, UCD SOM, Aurora, CO, USA; 3 University of Colorado Cancer Center, Aurora, CO, USA; 4 Department of Veterinary Clinical Sciences, University of Minnesota College of Veterinary Medicine, St. Paul, MN, USA; 5 Masonic Cancer Center, University of Minnesota, Minneapolis, MN, USA; 6 Department of Pharmacology, UCD SOM, Aurora, CO, USA; 7 Department of Veterinary Population Medicine, University of Minnesota College of Veterinary Medicine, St. Paul, MN, USA; 8 Department of Biostatistics, University of Alabama, Birmingham, Birmingham, AL, USA; 9 IHC Services, Smithville, TX, USA; 10 Department of Pediatrics, UCD SOM, Aurora, CO, 80045, USA; 11 Array BioPharma, CO 80301, USA; 12 Division of Hematology/Oncology, Department of Medicine, Medical University of South Carolina, Charleston, SC 29425, USA

## Abstract

**Background:**

The etiology of hemangiosarcoma remains incompletely understood. Its common occurrence in dogs suggests predisposing factors favor its development in this species. These factors could represent a constellation of heritable characteristics that promote transformation events and/or facilitate the establishment of a microenvironment that is conducive for survival of malignant blood vessel-forming cells. The hypothesis for this study was that characteristic molecular features distinguish hemangiosarcoma from non-malignant endothelial cells, and that such features are informative for the etiology of this disease.

**Methods:**

We first investigated mutations of VHL and Ras family genes that might drive hemangiosarcoma by sequencing tumor DNA and mRNA (cDNA). Protein expression was examined using immunostaining. Next, we evaluated genome-wide gene expression profiling using the Affymetrix Canine 2.0 platform as a global approach to test the hypothesis. Data were evaluated using routine bioinformatics and validation was done using quantitative real time RT-PCR.

**Results:**

Each of 10 tumor and four non-tumor samples analyzed had wild type sequences for these genes. At the genome wide level, hemangiosarcoma cells clustered separately from non-malignant endothelial cells based on a robust signature that included genes involved in inflammation, angiogenesis, adhesion, invasion, metabolism, cell cycle, signaling, and patterning. This signature did not simply reflect a cancer-associated angiogenic phenotype, as it also distinguished hemangiosarcoma from non-endothelial, moderately to highly angiogenic bone marrow-derived tumors (lymphoma, leukemia, osteosarcoma).

**Conclusions:**

The data show that inflammation and angiogenesis are important processes in the pathogenesis of vascular tumors, but a definitive ontogeny of the cells that give rise to these tumors remains to be established. The data do not yet distinguish whether functional or ontogenetic plasticity creates this phenotype, although they suggest that cells which give rise to hemangiosarcoma modulate their microenvironment to promote tumor growth and survival. We propose that the frequent occurrence of canine hemangiosarcoma in defined dog breeds, as well as its similarity to homologous tumors in humans, offers unique models to solve the dilemma of stem cell plasticity and whether angiogenic endothelial cells and hematopoietic cells originate from a single cell or from distinct progenitor cells.

## Background

The study of malignant soft tissue sarcomas that arise from, or resemble constituents of blood vessels in humans, including angiosarcomas (hemangiosarcomas and lymphangiosarcomas), Kaposi sarcomas, hemangioendotheliomas, and hemangiopericytomas, is complicated by their infrequent occurrence [1]. Despite their relatively low incidence, angiosarcomas are associated with more frequent metastasis and greater patient morbidity and mortality than other soft tissue sarcomas. The clinical significance of angiosarcomas is magnified because of their aggressive biological behavior and their association with medical or occupational exposures to ionizing radiation and a variety of industrial and agricultural chemical agents [1-3].

Other species also develop hemangiosarcomas. From a comparative perspective, hemangiosarcomas occur rarely in mice as a spontaneous disease, but the incidence is significantly increased in the B6C3F1 hybrid strain after exposure to various classes of pharmaceuticals, making these tumors a factor in risk assessment for drug development [2]. Dogs are the only species where idiopathic (spontaneous) hemangiosarcoma occurs commonly. This disease has been estimated to account for up to 7% of malignant canine tumors [4], which would roughly translate into >50,000 diagnoses per year in the United States. Regardless of species, treatment options for angiosarcoma and hemangiosarcoma are limited, and outcomes are generally unrewarding [5-7]. The standard of care in both humans and dogs includes surgery and adjuvant chemotherapy. The median and 5-year survival rates for human patients with angiosarcoma are reported to be approximately 2 to 2.5 years and 30%, respectively [1]. In dogs, the prognosis is equally grave: even though 10-15% of dogs with this disease survive 12 months or longer, most die within 3-months of their diagnosis [4]. Despite anecdotal success using immunotherapy, as well as novel chemotherapy and antiangiogenic strategies to treat canine hemangiosarcoma [8-13], the past 30 years have brought no improvements in survival for dogs with this disease [14].

The lack of effective treatments for humans and dogs with angiosarcoma and hemangiosarcoma is largely due to our incomplete understanding of the factors that promote the survival, growth, and metastases of these malignancies. Inflammation, hypoxia, and angiogenesis all might contribute to the pathogenesis of idiopathic hemangiosarcoma, or of hemangiosarcoma associated with exposure to non-genotoxic agents in each of the target species. The link between inflammation and cancer is becoming clearer, with macrophages and macrophage-derived cytokines playing a central role in modulating the tumor microenvironment to facilitate both tumor survival and metastasis [15-17]. Macrophage activation and local tissue hypoxia are central components of the proposed mechanism of action that drives hemangiosarcoma in rodents exposed to a diverse array of compounds such as 2-butoxyethanol, peroxisome proliferator-activated receptor (PPAR) agonists and pregabalin [2]. Parallels have been drawn between canine hemangiosarcoma cells and neoangiogenic endothelial cells in tumors [18,19]. Vessel formation in hemangiosarcoma resembles the morphology of imbalanced, chaotic growth and maturation of neoangiogenic vessels seen in cancer, which is at least partly driven by pro-angiogenic factors such as vascular endothelial growth factor-A (VEGF) [18,20]. In fact, hemangiosarcoma cells elaborate growth factors that promote angiogenesis, including not only VEGF, but also platelet-derived growth factor-β (PDGFβ), and basic fibroblast growth factor (bFGF) *in vitro *[14,18,19,21]. Signaling by each of these growth factors is partly dependent on activation of the phosphoinositide 3-kinase (PI3K) pathway, providing a possible connection between the processes of inflammation, hypoxia, and angiogenesis in the pathogenesis of hemangiosarcoma [22]. In this regard, mutations of the PI3K antagonist, PTEN, are common in canine hemangiosarcoma; however they are restricted to the C-terminal domain and do not affect the phosphorylation of Akt that occurs downstream from PI3K signaling [23]. While it is possible that mutations in the C-terminal domain reduce the stability of PTEN [24] or increase motility, and hence a cell's invasive potential [25,26], the precise effects of these mutations in canine hemangiosarcoma remain unclear. The genetic basis of abnormal patterns of growth and signaling require further characterization.

Mutational events have been documented in sporadic angiosarcomas of humans and hemangiosarcomas of mice and dogs, including cancer-associated genes such as PTEN, Ras, VHL, p53, and connexin, [27-34]. In the case of canine hemangiosarcoma, PTEN mutations did not fully explain the increased levels of VEGF or other growth factors [18,23], prompting additional assessment of potential roles for mutations that inactivate VHL or that activate Ras, as both can lead to elevated VEGF production. Yet, another possibility was that non-malignant cells are responsible for VEGF production in canine hemangiosarcoma [35], especially since co-existence of tumor cells with inflammatory cells is a common feature of this disease, and in some cases, the inflammatory cells may provide the principal source of VEGF [23]. In this scenario, VEGF-producing inflammatory cells could be reactive leukocytes incited by pathologic effects of tumor (e.g., tissue destruction), or macrophages and myeloid cells that are intrinsic components of the tumor microenvironment [17,35]. A third possibility is that hemangiosarcomas originate from a multipotent bone marrow progenitor that can differentiate along the myeloid lineage [36,37], and these cells thus could reflect the ontogeny of the malignant cells and their plasticity to differentiate into multiple cell types.

Here, we show in an isolated *in vitro *system that expression of genes involved in inflammation, angiogenesis, adhesion, invasion, metabolism, cell cycle, signaling, and patterning can distinguish hemangiosarcoma cells from non-malignant endothelial cells. While the data do not distinguish whether functional or ontogenetic plasticity creates this phenotype, they suggest the factors that hemangiosarcoma cells use to communicate with their microenvironment are distinct from those used by non-malignant endothelial cells. To our knowledge, this is the first report that establishes differences between hemangiosarcoma and non-malignant endothelial cells in any species, highlighting biochemical and metabolic pathways that may be amenable to therapeutic targeting for this disease.

## Methods

### Samples

Between 2000 and 2005 we received samples from 63 pet dogs with pathologically confirmed hemangiosarcoma (N = 58) or pathologically confirmed splenic hematoma (N = 5). Eighteen tumors and four splenic hematomas included fresh, viable tissue as part of the submission, while the remainder included only fixed or frozen tissue. For each sample set that included viable tissues, we cultured a portion of tissue under conditions that favored growth of endothelial cells to derive cultured cell lines from tumors or low passage cultures from the splenic hematomas [18,23,36]. We derived such cultures successfully from 10/18 hemangiosarcomas and from four of five splenic hematomas, which were used for the analyses described in this study (see Additional File 1, Table S1). The morphology of the resulting cell lines was variable [18], but each expressed antigens that are characteristically expressed by blood vessel-forming cells [18,23,36]. To ensure there were no hidden biases in the sample population, we examined descriptive statistics between the 14 dogs from which we established cells in culture and from the 49 dogs for which we did not establish cell lines. There were no significant differences between the two groups when comparing geographic origin, gender, age at diagnosis, anatomic location of the primary tumor or lesion, number of dogs treated, or outcome. The characteristics of both populations also were similar to those described elsewhere [38]. For tumors, cells from earliest passages available were used for the experiments. High molecular weight genomic DNA and total RNA were isolated as described [39,40]. Only two of the dogs whose samples were used for the microarray experiments (Frog and Journey) were related within five generations, and they were separated by three generations (Frog was Journey's paternal "great aunt"), reducing the likelihood of lineage bias. To confirm the data from the initial 10 hemangiosarcoma samples and to assess how the results from cell lines correlated with data from intact tissues, we established four additional cell lines from two dogs with hemangiosarcoma and used these, as well as three independent whole tissue samples in microarray experiments. One cell line (Emma-HSA) was previously reported [40]. Three additional lines, called Jack-liver HSA, Jack-spleen HSA, and Jack-heart HSA were established form one dog with metastatic hemangiosarcoma (spleen, liver, and heart). The tissue samples all were from splenic tumors where sufficient, high quality snap-frozen and cryopreserved material was available.

Peripheral blood samples were obtained from dogs with hemangiosarcoma or other tumors by a licensed veterinarian or an animal health practitioner as part of medically necessary (biopsy) procedures prior to initiating therapy or at the time of necropsy. Samples from healthy dogs obtained as part of routine diagnostic or well-health procedures were used as controls. Non-hemangiosarcoma diagnoses included non-Hodgkin lymphoma, leukemia, and osteosarcoma. Every sample was obtained with owner consent through protocols reviewed by appropriate Institutional Animal Care and Use Committees.

### Assessment of VHL and Ras gene expression

The procedures used to clone and confirm the sequence of canine VHL are described in the Supplementary Methods (see Additional File 2; Genbank ID numbers GU563722 and GU563723). The nucleotide sequence and the translated amino acid sequence for canine VHL are in Additional File 3, Figure S1. The conditions for RT-PCR to amplify the complete coding sequence from the ATG start codon to the TGA stop codon included forward primer, CGTTGTCTAGGCTCCGGG, reverse primer, GGCTGAGACTCAGGAGTGC, and annealing temperature of 60°C. We used RT-PCR conditions described previously to amplify the complete coding sequence for canine N-Ras, K-Ras, and H-Ras [41].

### Gene expression profiling

Approximately 2.5 μg of RNA were labeled using the Affymetrix labeling protocol (Affymetrix, Santa Clara, CA, USA), with cRNA samples hybridized to Affymetrix Canine_2 gene expression chips as described [40]. One sample was done in duplicate (2 chips) to control for batch effects. The concordance between the data from duplicate chips was >97%. The theoretical principles and the empirical observations used to support the sample size for these experiments a priori were essentially as we described before [40]. The PowerAtlas (http://www.poweratlas.org, ref. [42]) allowed us to obtain an empirical estimate that the imbalanced sample sets used for these experiments with 10 hemangiosarcomas and three splenic hematomas should provide >85% power at α = 0.05 to identify true positives, although the power to identify true negatives could be lower. Bioinformatic analysis followed previously described protocols, including normalization, filtering, assignments of functional ontogeny, and gene set enrichment for pathway identification [40]. All data are available through the Gene Expression Omnibus http://www.ncbi.nlm.nih.gov/geo/ with access number GSE22129 or by searching the term "hemangiosarcoma". These data also are combined with those from our previous study [40] in a GEO SuperSeries (accession number GSE23760). Ingenuity Pathway Analysis (IPA) software v8.6 (Ingenuity Systems, Redwood City, CA, USA) was used to define functions and canonical pathways of genes identified by GSEA using BH multiple testing corrections to assess significance.

### Real-time quantitative RT-PCR (RT-qPCR)

RT-qPCR for TIMP-1, PLZF, and FN-1 was performed at 50°C for 2 min, 95°C for 10 min, and then 40 cycles of 95°C for 15 s and 60°C for 1 min per cycle. Forward primers, reverse primers, and Taqman probes (5' to 3' orientation) were: for TIMP-1 GAGAGCGTCTGCGGATACTTG, TCCGGCGACCAGAAACTC, and ACAGGTCCCAGAACC, for PLZF ACGGACATGGCCGTCTTC, CGCTCTGCGCCTGGAA, and TGCTGTGTGGGAAGC, and for FN-1 GCCAGCCCCTGATTGGA, CCAGCGGTGGCAGTGAAC, and CCCAGTCCACAGGTATA. Each reaction was done in triplicate and normalized to endogenous 18 S gene using Taqman Fast Reagent Starter Kit (ABI).

### Immunostaining

Immunohistochemistry on formalin-fixed and paraffin-embedded tumor samples and immunocytochemistry on freshly grown cells were done at IHC Services (Smithville, TX, USA) as described [23,43]. Antibodies were selected based on known cross-reactivity against the canine protein or on their generation using highly conserved regions of the protein as immunogens. Specifically, we used antibodies against VHL (antibody G-7, Santa Cruz Biotechnology, Santa Cruz, CA, USA), pan-Ras (all family members, antibody C-4, Santa Cruz Biotechnology), Erk 1/2, (antibody MK1, Santa Cruz Biotechnology), phospho-Erk 1/2 (antibody 12D4, Santa Cruz Biotechnology), and platelet-derived growth factor receptor-β (PDGFRβ, antibody P20, Santa Cruz Biotechnology) for immunostaining. Expression of HIF-1α was verified by immunoblotting as described [23,40] using an anti- HIF-1α antibody from Novus Biologicals (Littleton, CO, USA).

## Results

### Mutations of VHL and Ras genes are infrequent in canine hemangiosarcoma

**Figure 1:**
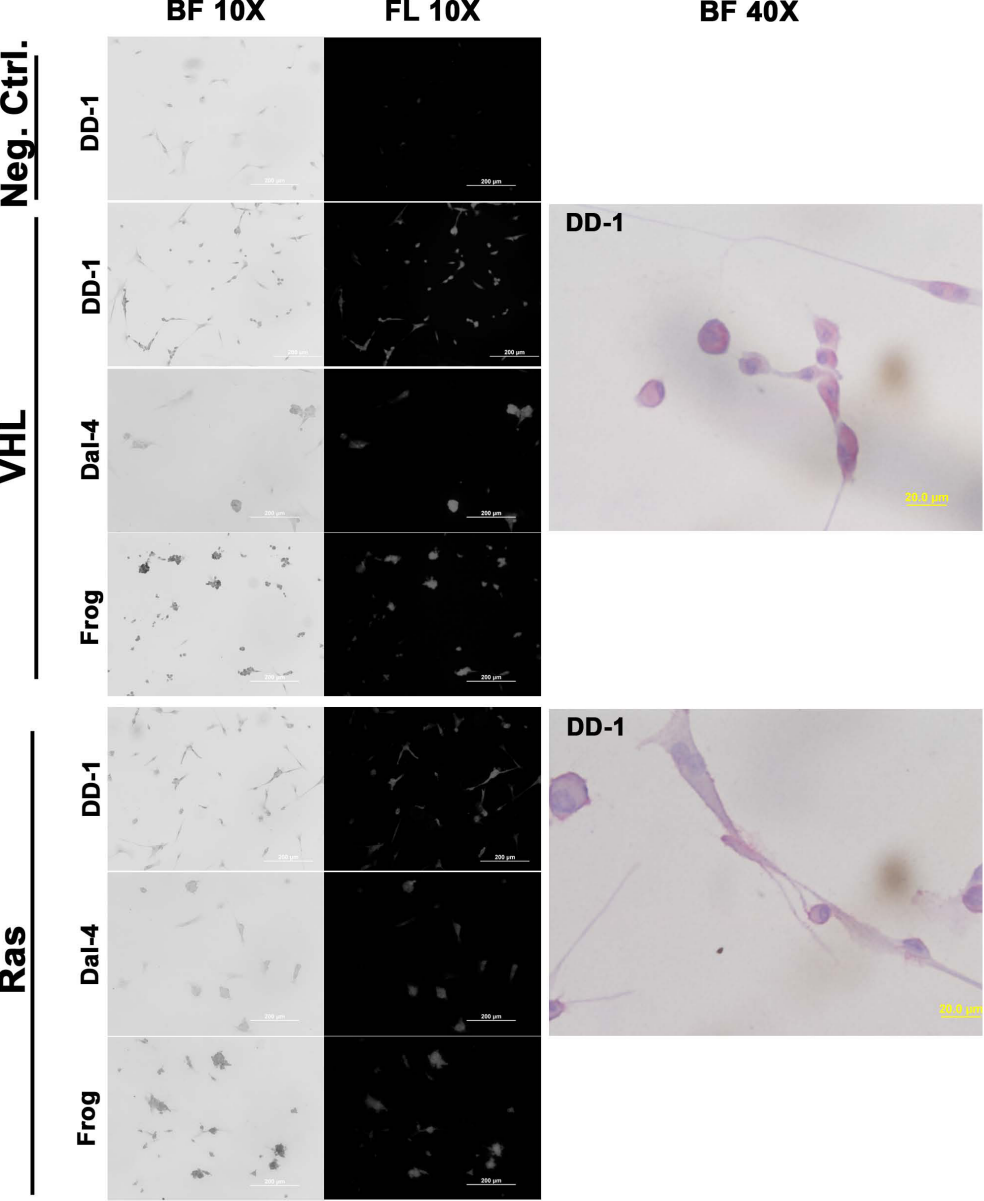
**Expression of VHL and Ras proteins in canine hemangiosarcoma cell lines**. DD-1, Dal-4, and Frog canine hemangiosarcoma cell lines were cultured in chamber slides and stained with an irrelevant control antibody, or with antibodies against VHL or pan-Ras as indicated on the left. Cells were visualized using bright field microscopy at low magnification (10× objective, BF 10×) and staining in the same fields was visualized using epifluorescence (FL 10×). Bars = 200 μm. Right panels show photomicrographs of DD-1 cells under bright field illumination at high magnification (40 × objective, BF 40×) to illustrate the localization of VHL to the cytoplasm and the localization of Ras predominantly to the inner plasma membrane (red staining). Bars = 20 μm.

**Figure 2:**
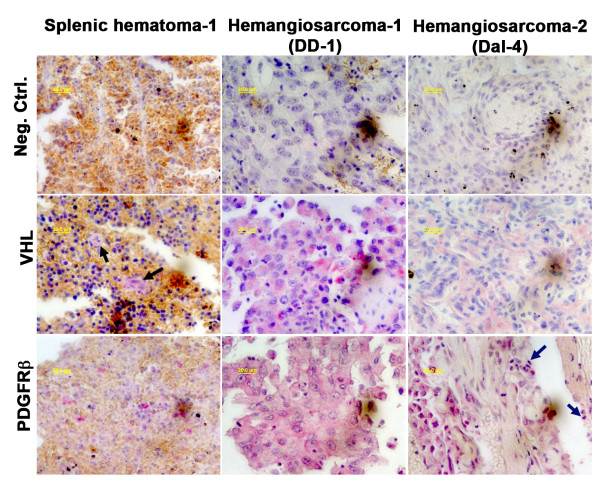
**Expression of VHL and PDGFRβ proteins in canine splenic hematoma and in canine hemangiosarcoma tissues**. Serial 5-μm sections from paraffin-embedded splenic hematomas or hemangiosarcomas were stained with an irrelevant control antibody, or with antibodies against VHL or PDGFRβ as indicated on the left. Photomicrographs represent similar or contiguous regions within each tissue at 640× magnification. Two samples of splenic hematomas and eight samples of canine hemangiosarcoma were stained for VHL. Both splenic hematomas and five hemangiosarcomas were stained for PDGFRβ. One of the splenic hematomas and two hemangiosarcomas (the tumors used to derive the DD-1 and Dal-4 cell lines) are shown to illustrate the observed patterns. Expression of relevant antigens is indicated by red staining. VHL staining in the splenic hematomas was restricted to blood vessel lining cells (black arrows), whereas in the hemangiosarcomas, VHL staining was seen diffusely in the tumor cells, but not in associated inflammatory cells. In contrast, PDGFRβ staining was seen in blood vessel lining cells in the splenic hematomas, but was seen both in tumor cells and in associated inflammatory cells in the hemangiosarcomas (blue arrows). Bars = 20 μm

We sequenced the complete coding domains for VHL, N-Ras, K-Ras, and H-Ras from 10 canine hemangiosarcoma cell lines and from four lines derived from splenic hematomas. These genes are infrequently mutated in angiosarcomas of humans and hemangiosarcomas of rodents. Mutations in these genes appear to be similarly infrequent in canine hemangiosarcoma, as each of the tumor samples, as well as the splenic hematoma samples we evaluated in this study had wild-type sequence for VHL and for the three Ras family genes. Expression of VHL and Ras proteins was confirmed by immunostaining in the cell lines (Figure 1), and VHL expression also was verified in eight archival samples representing original tumors used to derive the cell lines by immunohistochemistry (Figure 2). VHL expression in the splenic hematomas was restricted to endothelial cells (shown by black arrows in Figure 2), whereas the protein was uniformly detectable in hemangiosarcoma cells, but not in stromal or inflammatory cells, in the tumor sections. Hemangiosarcoma cell lines expressed HIF1α at approximately comparable levels to those seen in human ACHN renal cell carcinoma cells with wild type VHL (data not shown), suggesting there was no abnormal accumulation of this protein. Immunostaining of Erk1 and Erk2 proteins, which operate downstream from Ras, was barely detectable in each of three cell lines examined, and of the three canine hemangiosarcomas cell lines, only "Frog" showed evidence of constitutively active Erk1 and Erk2 based on positive staining by the presence anti-phospho-Erk antibody (Thr 202 and Tyr 204). Phospho-Erk1/2 proteins in this cell line were restricted to the cell membrane in areas of cell-to-cell contact at the periphery of large multicellular clusters (see Additional File 4, Figure S2), and were not seen in individualized cells or in cells forming smaller clusters, suggesting activation was probably mediated by intercellular signaling. These findings provide further evidence that abnormal activation of the VHL and Ras pathways is not a common occurrence in canine hemangiosarcoma. Indeed, the probability that we would observe no mutations if the sample size had a binomial distribution and the mutation rate were high as 0.28 is approximately 1%. Therefore, we conclude that the frequency of VHL or Ras mutations in sporadic canine hemangiosarcoma is unlikely to exceed 30%.

### Gene expression analysis segregates canine hemangiosarcoma cells from proliferating endothelial cells of splenic hematoma

We hypothesized that hemangiosarcoma cells would be distinguishable from non-malignant proliferating endothelial cells, such as those found in splenic hematoma, based on gene expression profiles. We further hypothesized that these profiles would be informative for the pathogenesis of this disease. We chose to evaluate gene expression profiles in the low passage cultured cells, which we surmised would provide a more homogeneous population than fresh tumor samples by excluding non-malignant stromal components and inflammatory cells, mitigate genes associated with phenotypic endothelial commitment, and diminish the influence of differential proliferative signatures. Nonetheless, it was likely that hemangiosarcoma cells would preserve signatures associated with incomplete differentiation.

**Figure 3:**
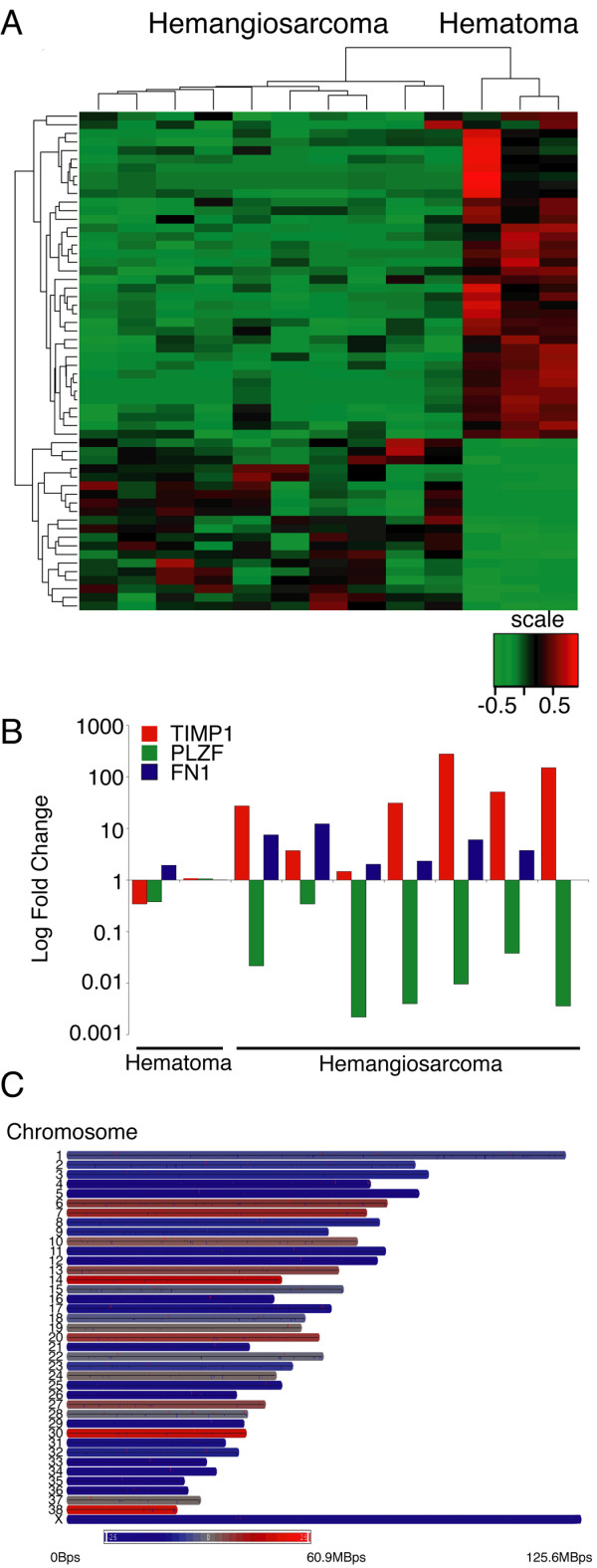
**Canine hemangiosarcoma cells segregate from non-malignant splenic hematoma cells via their gene expression profile**. (A) Hierarchical clustering and heat map of differentially expressed genes in 10 hemangiosarcoma samples versus three splenic hematoma samples. Increasing red intensity indicates increased gene expression and increasing green intensity indicates decreased gene expression as shown in the scale bar. The scale (-1 to +1) reflects variation in intensity from the mean (0) and not fold-change. Fold-change differences are shown in Table S2. (B) Quantitative expression and graphical representation of 2 genes shown in Figure 2A (TIMP-1 and PLZF) and 1 additional gene (FN-1) that were differentially expressed between hemangiosarcoma and splenic hematoma cells. Samples were evaluated for gene expression changes by RT-qPCR, normalized to the endogenous 18 S gene. One sample originating from a dog with splenic hematoma was set to the unitary value (1.0) and used as the calibrator; gene expression is presented as the log fold-change compared to the calibrator. (C) Genes whose expression was significantly different (p <0.05) between hemangiosarcomas and splenic hematomas based on analysis of variance of the complete filtered lists were plotted according to their cytogenetic location on the 38 canine autosomes and the X chromosome. The color intensity for each chromosome (red-over represented to gray to blue-under represented) represents the sum of all changes.

RNA from ten hemangiosarcomas and from three splenic hematomas was processed and hybridized to Canine_2 Affymetrix chips. After data were normalized and filtered, a list of 14,028 genes remained that allowed comparisons between the two sample sets. Hierarchical clustering from this list separated the samples into two main groups, consisting of hemangiosarcoma and splenic hematoma (Figure 3A). Within the hemangiosarcoma group, two major subgroups also separated golden retrievers from non-golden retrievers [40]. When the False Discovery Rate (FDR) was set to <0.1, we uncovered a 58-probe signature representing 30 genes, four predicted genes, and nine transcribed loci, which was differentially expressed between both groups. A heatmap using these differentially (over- or under-) expressed probes is shown in Figure 3a, and the genes are listed in the order they appear in the heatmap in Table S2 (see Additional File 5). A functional annotation is provided in Table S3 (see Additional File 6).

Microarray data were validated by rigorous statistical tests; however, we verified changes by RT-qPCR of TIMP-1 and PLZF, two genes expressed abnormally in other cancers [44,45]. TIMP-1 expression was estimated to be, on average, 7 fold higher in hemangiosarcomas than in splenic hematomas based on microarray data, whereas RT-qPCR showed changes as large as +800 fold (Figure 3B). In the case of PLZF, expression in hemangiosarcoma was estimated to be, on average, 10.5 fold lower in hemangiosarcomas than in splenic hematomas based on microarray data, and indeed, PLZF levels were 10 to 500 fold lower in hemangiosarcoma samples based on RT-qPCR.

According to the Power Atlas prediction, the likelihood that genes on our list were true positives was high, but the expected discovery rate might have been low. We selected Fibronectin (FN-1), a gene involved in wound healing, blood coagulation, and cancer metastasis [46], as a representative candidate that showed significantly different expression in hemangiosarcoma and splenic hematoma, but did not meet the initial FDR criteria. FN-1 expression in hemangiosarcoma samples was estimated to be, on average, 2 fold higher in hemangiosarcomas than in splenic hematomas based on microarray data, and RT-qPCR confirmed expression was increased by 2 to 12 fold (Figure 3B).

When all of the genes showing significantly different levels of expression were arranged according to their cytogenetic location, hemangiosarcoma genomes showed remarkable underexpression (commonly absent) compared to the splenic hematoma genomes (Figure 3C). These changes are similar to those reported for various other tumors [47] and could be caused by deletions, translocations, or epigenetic changes such as methylation. These data also show the sensitivity of this approach. There were 3 females in the hemangiosarcoma group (30%) and 2 females in the splenic hematoma group (67%). As one would predict from these ratios, genes in the X chromosome appeared to be consistently underexpressed in the hemangiosarcoma group.

### Canine hemangiosarcoma gene signatures are distinct and do not reflect simply malignant transformation

**Figure 4:**
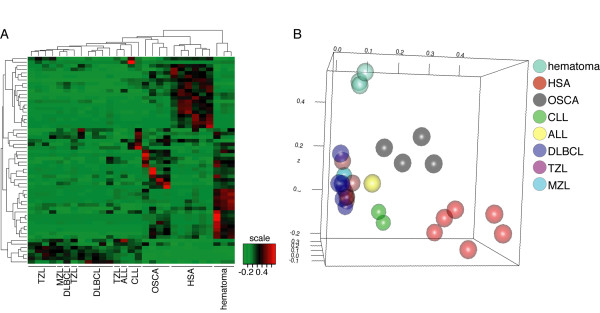
**Hemangiosarcoma is distinguishable from both non-malignant and other malignant tumors**. (A). Hierarchical clustering of tumor samples or non-malignant lesions (hematoma) from golden retrievers. Tumor samples were from osteosarcoma cell lines (OSCA), primary leukemia (ALL-Acute lymphoblastic leukemia or CLL-Chronic lymphocytic leukemia) or non-Hodgkin lymphoma (diffuse large B-cell lymphoma, marginal zone lymphoma, or T-zone lymphoma) cells, or hemangiosarcoma cell lines (HSA). Hierarchical clustering was done using the restricted probe list from Table S2. (B) Principal component analysis (PCA) of tumor samples and non-malignant lesions described in (A), except analysis was done using all data points.

To establish whether these changes were specific to hemangiosarcoma, or if they were simply associated with any malignancy, we compared array data from six hemangiosarcoma samples with data from five osteosarcomas, three leukemias, and 13 non-Hodgkin lymphomas. We used samples from golden retrievers exclusively as a way to minimize the potential bias that breed (genetic background) might introduce on gene expression signatures [40,48]. This comparison yielded 1,092 probes with FDR < 0.001, suggesting that indeed, hemangiosarcomas have unique gene expression signatures. Specifically, if we limited the analysis to the 58 differentially expressed probes from the analysis shown in Figure 3A and Table S2, hemangiosarcomas were readily segregated from the other tumors and from the non-malignant endothelial cells (Figure 4A), suggesting at least ~30 genes show relatively unique and consistent patterns of coordinate expression in hemangiosarcoma cells. Moreover, hemangiosarcoma samples also were readily distinguishable from the non-hemangiosarcoma samples by principal component analysis using unfiltered data (Figure 4B).

Genes that were expressed at significantly higher levels in hemangiosarcomas than in non-hemangiosarcoma tumors (osteosarcoma, non-Hodgkin lymphoma, and leukemia) included VEGFA, TIMP-1, FN-1, ADAM9, PDGFC, MMP14, TNFα, and acid ceramidase, which also were more highly expressed in hemangiosarcomas than splenic hematomas. This gene signature suggested inflammatory and angiogenic pathways play a significant role in the pathogenesis of hemangiosarcoma. Moreover, hemangiosarcomas might preferentially utilize survival pathways involving ceramide signaling, which may be less commonly used by other tumors.

### Pathway analysis provides insight into hemangiosarcoma tumor biology

Hierarchical clustering and other similar analyses are informative to determine overall similarity between samples and to separate samples into defined groups based on molecular signatures. They do not, however, provide definitive information regarding how the genes may be functionally related and thus contribute to the biology of the tumors. Several bioinformatic approaches have been developed to infer such functional relationships, including gene ontology (ONTO) and gene set enrichment analysis (GSEA). We predicted this approach would identify abnormally expressed genes concentrated in pathways that reflect the origin and progression of this disease. We used the ONTO/express software to identify functional pathways for each gene in Table S2. These pathways are commonly dysregulated in cancer; however, ONTO analysis failed to identify pathways that would converge on one or a few recurrent abnormalities.

**Figure 5:**
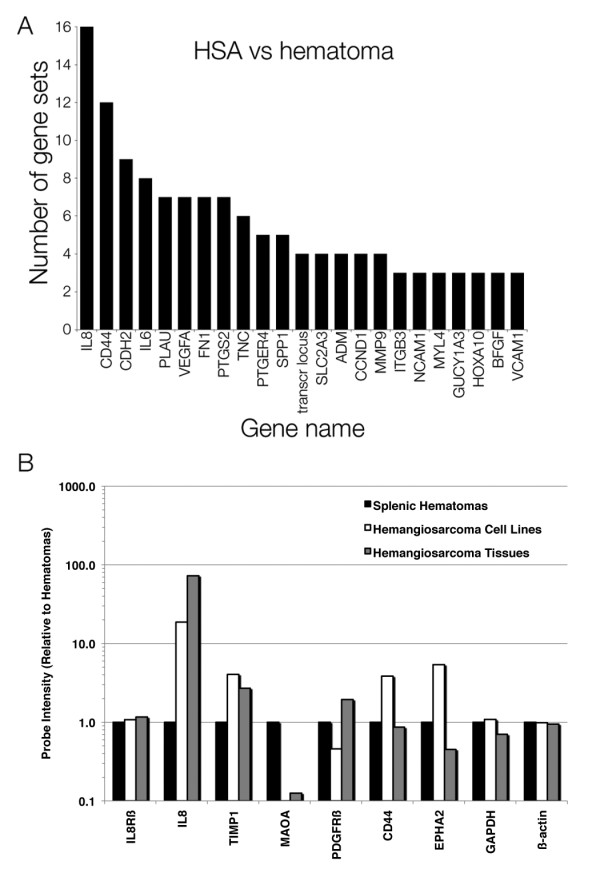
**Gene set enrichment analysis validates the hypothesis that the hemangiosarcoma gene set is involved in hypoxia, inflammation, and cancer**. (A) Bar graph representing the number of gene sets that were enriched in hemangiosarcoma samples versus splenic hematoma samples. Each of 23 genes on the x-axis was present in the number of gene sets indicated on the y-axis (of 30 where FDR < 0.05). (B) Bar graph representing the direction and magnitude of change in expression for six representative genes (IL8, TIMP1, MAO, PDGFRβ, CD44, EPHA2), one invariant control (IL8Rβ) and two housekeeping controls (GAPDH, β-actin) relative to the expression in splenic hematomas. Data for each group (three splenic hematomas, 14 hemangiosarcoma cell lines, and three hemangiosarcoma tissues) passed quality assurance using Affymetrix algorithms provided in GeneData Expressionist Refiner. Probe signal levels were quantile-normalized and summarized using the GeneChip-Robust Multichip Averaging (GC-RMA) algorithm. Normalized files were imported into GeneData Expressionist Analyst so average expression values for each group, based on multiple-probe hybridization data, could be used in the comparisons.

GSEA is a robust computational tool that can uncover subtle alterations in complex diseases by utilizing expression data to characterize gene signatures within pathways. Pathways that defined cellular processes underlying hypoxia, cancer, or inflammation were highly enriched in hemangiosarcoma (Table 1). We used the leading edge subset to compare genes from each pathway to 30 other pathways that showed enrichment a FDR < 0.05. One hundred and fifty-seven genes were present in at least one pathway, 31 were recurrently present in at least two pathways, and 23 were in at least three pathways. The results from these top 23 genes show that IL8 was enriched in 16 of the 30 pathways, CD44 in 12, CDH2 in nine, IL6 in eight, VEGFA, PLAU, PTGS2 and FN-1 in seven each, TNC in six, PTGER4 and SSP1 in five, SLC2A3, ADM, CCND1, and MMP1 in four, and several genes, including VCAM1 in three (Figure 5A).

**Table 1:** Gene set enrichment analysis predicts pathways involved in inflammation, cancer, and hypoxia are important for hemangiosarcoma^a^

Gene set	Description	ES	NES	FDR
MENSE_HYPOXIA_UP	Hypoxia induced genes in HeLa and astrocytes	0.82	2.32	0.000
BRENTANI_CELL_ADHESION	Cancer related genes involved in cell adhesion and metalloproteinases	0.67	2.15	0.005
LINDSTEDT_DEND_8H_VS_48H_UP	Genes upregulated in stimulated Dendritic cells	0.75	2.15	0.003
HYPOXIA_REVIEW	Genes known to be induced by hypoxia	0.62	2.12	0.003
KNUDSEN_PMNS_UP	Genes up-regulated in PMNs upon migration to skin lesions	0.73	2.10	0.003
CHIARETTI_T_ALL	Genes overexpressed in leukemia	0.61	2.10	0.002

^a^The filtered gene list from hemangiosarcoma vs. non-malignant hematomas were compared using the GSEA software. ES (Enrichment Score) is a value that represents how well the gene set is enriched within the selected gene list. NES (normalized enrichment score) corrects the ES for differences in gene set size and can be used to compare across gene sets. A high ES or NES indicates that gene set is highly enriched within our gene list. The lists shown are those gene sets with an NES of 2.10 or higher.

The recurrent enrichment of proinflammatory cytokines, adhesion molecules, and angiogenic factors in these cells suggests that modulation of the microenvironment is an essential feature of hemangiosarcoma and highlights genes that, when treated as a group, may be prognostically significant and amenable to therapeutic intervention. We thus examined if the relation of 30 recurrently enriched genes (30 of the 31 genes present in two or more GSEA pathways were annotated) would remain when analyzed using Ingenuity Pathway Analyses (IPA, Table 2). IPA highlighted functional pathways associated with malignancy (21/30 genes), proliferation and survival (22/30 genes), migration, metastasis, and adhesion (22/30 genes), vascular biology and endothelial ontogeny (16/30 genes), and inflammation (14/30 genes). The top 10 canonical pathways (-log BH p values >3) were similarly all associated with malignancy and inflammation. Figure S3 (see Additional File 7) illustrates the relationship of six recurrently enriched molecules (IL8, CCND1/Cyclin D1, MMP9, VEGF, VCAM1, and PTGS2/Cox-2) in one of these canonical pathways (IL8 signaling), which mediates both angiogenesis and inflammation.

**Table 2:** Function annotation from IPA for 23 recurrently enriched genes identified by GSEA^a^

*Function Annotation*	*B-H p-value*	*Molecules*	*# Molecules*
Proliferation of normal cells	1.35E-15	ADM, CCND1, CD44, CDH2, FGF2, FN1, GUCY1A3, HOXA10, IL6, IL8, IL12A, ITGB3, MMP9, NCAM1, PLAU, PTGS2, S1PR1, SPP1, SPTBN1, TGFBR2, TNC, VCAM1, VEGFA	23
Migration of normal cells	1.77E-17	ADM, CCND1, CD44, CDH2, FGF2, FN1, GUCY1A3, IL6, IL8, IL12A, ITGB3, MMP9, NCAM1, PLAU, PTGER4, PTGS2, S1PR1, SPP1, TGFBR2, TNC, VCAM1, VEGFA	22
Malignant tumor	3.18E-10	CCND1, CD44, CDH2, FGF2, FN1, IL6, IL8, IL12A, ITGB3, MMP9, NCAM1, PLAU, PTGER4, PTGS2, S1PR1, SPP1, SPTBN1, TGFBR2, TNC, VCAM1, VEGFA	21
Apoptosis	9.50E-10	ADM, CCND1, CD44, CDH2, FGF2, FN1, IL6, IL8, IL12A, ITGB3, MMP9, NCAM1, PLAU, PTGER4, PTGS2, S1PR1, SLC2A3, SPP1, TGFBR2, TNC, VEGFA	21
Development of blood vessel	3.09E-14	CCND1, CDH2, FGF2, FN1, IL6, IL8, IL12A, ITGB3, MMP9, PLAU, PTGER4, PTGS2, S1PR1, TGFBR2, VCAM1, VEGFA	16
Angiogenesis	3.09E-14	CCND1, FGF2, FN1, IL6, IL8, IL12A, ITGB3, MMP9, PLAU, PTGER4, PTGS2, S1PR1, TGFBR2, VCAM1, VEGFA	15
Adhesion of normal cells	2.82E-14	CCND1, CD44, CDH2, FGF2, FN1, IL6, IL8, ITGB3, NCAM1, PLAU, SPP1, TGFBR2, TNC, VCAM1, VEGFA	15
Proliferation of blood cells	3.62E-09	CD44, FGF2, FN1, HOXA10, IL6, IL8, IL12A, ITGB3, MMP9, PTGS2, SPP1, TGFBR2, VCAM1	13
Metastasis	1.11E-12	CD44, CDH2, FGF2, IL6, IL12A, ITGB3, MMP9, NCAM1, PTGS2, SPP1, TGFBR2, VEGFA	12
Inflammatory response	7.51E-09	CCND1, CD44, FN1, IL6, IL8, MMP9, PLAU, PTGER4, PTGS2, S1PR1, SPP1, VEGFA	12
Inflammation	1.01E-09	CD44, FGF2, IL6, IL8, IL12A, MMP9, PTGER4, PTGS2, SPP1, TGFBR2, VEGFA	11
Infiltration of cells	3.00E-10	CD44, FN1, IL6, IL8, IL12A, ITGB3, MMP9, SPP1, VCAM1, VEGFA	10

^a^Annotated genes recurrently enriched in GSEA (> 2 pathways) were analyzed using IPA. Four hundred pathways were identified with a B-H p value < 1 × 10^-5^. Virtually all of them were categorized as malignancy, proliferation and survival, vascular processes, and inflammation. The table shows 12 representative pathways with a B-H p value <1 × 10^-8^, organized according to number of genes included.

Finally, to establish the relevance of these molecules and pathways in vivo, we compared mean expression levels of 11 annotated genes that were differentially expressed, and/or differentially enriched, among the three cultured hematoma samples, 14 hemangiosarcoma cell lines (the 10 original lines used in the experiment and four newly established lines), and three whole tissue samples. We surmised that differences in recurrently enriched genes where differences in expression were relatively low would be the most robust indicators of whether these signatures were relevant to the biology of hemangiosarcoma or simply altered as a function of cell culture or anatomical site of origin.

Data from six representative genes are shown in Figure 5B. In addition, expression levels of IL8Rβ are shown as an example of an invariant control (no difference in between cultured non-malignant endothelial cells and hemangiosarcoma cells), and GAPDH and β-actin were used as housekeeping controls to confirm that the normalization strategy was valid. Expression of IL8, TIMP1, and MAOA in whole tissue samples corroborated the data from cultured cells. A similar trend was apparent for the RTK-like orphan receptor, Oncostatin M receptor, and Kinesin family member 5C. On the other hand, expression of PDGFRβ, CD44, and EPHA2 in hemangiosarcoma cell lines was not predictive for the expression of these proteins in tumor tissues (Figure 5B), and a similar trend was observed for Neuropilin 1 and v-Myc. We thus investigated the possibility that these differences might be attributable to stromal elements in the tumors. As illustrated by in Figure 2, PDGFRβ was expressed predominantly by blood vessel lining cells in splenic hematomas, whereas in the tumors, it was expressed generally at lower intensity in the hemangiosarcoma cells, but it also was expressed in infiltrating inflammatory cells (blue arrows). This suggests that gene expression by stromal cells contributes to genome-wide signatures from whole tissues; hence, supporting the rationale to use enriched cell cultures and cell lines as an approach to mitigate signatures from non-tumor components.

## Discussion

The mechanisms underlying the origin and progression of hemangiosarcoma remain unclear. In humans, angiosarcoma can be associated with exposure to DNA-damaging agents or, in the case of Kaposi sarcoma, with infection by HHV8 in immunosuppressed patients [1,49]. In mice, hemangiosarcoma can develop in susceptible strains treated with both genotoxic and non-genotoxic agents [2]. In dogs, however, the disease occurs sporadically (not as a heritable condition) and with alarming frequency in the absence of known mutagens; a canine gamma herpes virus also has not been characterized [50]. Indeed, preliminary experiments using well established methods to detect gamma herpes viruses [51] yielded no amplification products when applied to these canine hemangiosarcoma samples (M. Duckett and M. Cannon, unpublished results), suggesting the etiology of canine hemangiosarcoma does not involve infection by a gamma herpes virus.

Angiosarcoma in humans and hemangiosarcoma in dogs are rapidly progressive diseases that are poorly responsive to conventional therapy. An improved understanding of their pathogenesis is needed to develop effective strategies for prevention and treatment. Our data suggest that inflammation and angiogenesis (defined by enrichment of cytokines and adhesion molecules that may be downstream effectors of a single molecule, like IL8, IL6, or IL1, as well as by robust upregulation of VEGF, MMPs and TIMPs, PDGF and PDGFRs, and others) are two general processes that are central to the pathogenesis of canine hemangiosarcoma.

Here, we first tested the hypothesis that known genes that regulate VEGF, including VHL and members of the Ras family were targets of mutation in canine hemangiosarcoma. We previously showed that the PTEN/Akt pathway that appears to be essential in other vascular tumor models is intact in hemangiosarcoma cells [23]. Our observations that every hemangiosarcoma sample tested had wild type sequence for VHL, N-Ras, K-Ras, and H-Ras, no significant elevations of HIF1α, and no constitutive activation of Erk1 and Erk2 suggest that dysregulated VEGF production and the aggressive proliferation seen in these tumors are probably mediated by mechanisms that are independent from abnormalities of VHL and Ras genes. Recently, Pressler reported similar findings with regard to mutations of VHL in sporadic canine renal cell carcinoma [52]. When considered along with the estimate that solid tumors from humans (colon and breast carcinomas) carry an average of ~100 gene mutations, these results suggest the probability to identify recurrent abnormalities by candidate gene approaches based on lineage similarity or dysregulation of a single known pathway is low. Indeed, even the recurrent mutations of the C-terminal domain of PTEN that we characterized previously were unlikely to be singularly responsible for the behavior of hemangiosarcoma or tractable for therapy. We hence tested the hypothesis that canine hemangiosarcoma would show characteristic gene expression profiles that would be informative for etiology and progression.

Genes that regulate cellular metabolism, cell cycle and cell signaling, cell-cell interactions, survival and apoptosis, angiogenesis, transcription, and the immune response were among those dysregulated in hemangiosarcoma cells when compared to non-malignant proliferating endothelial cells. Our data specifically highlight pathways that are important in response to hypoxia or angiogenesis, malignant transformation, and inflammation. However, altered expression of genes within these functional categories could describe virtually any solid tumor (where cyclins, glucose transporters, and other genes associated with the mitotic cell cycle commonly show elevated expression) from its normal counterparts. For example, we recently showed that elevated expression of CDKN1a (p21) confers chemoresistance to renal cell carcinomas, which share common hypoxia-induced, pro-angiogenic signatures with vascular tumors [53]. The presence of hypoxia-inducible genes, including HGF, VEGF, bFGF, ADM, and PTGER4 was especially predictable [54,55]; in most tumors, cells become hypoxic and upregulate genes that promote blood vessel outgrowth and that control metabolic processes such as vasodilation that can make cells normoxic. Clonal evolution in the tumor, and perhaps the environment in cell culture might favor selection of cells that upregulate such hypoxia response genes, although expression of these genes might be inherent to tumors of blood vessel forming cells.

Recently, Antonescu et al reported that human angiosarcomas have unique gene expression profiles when compared to other soft tissue sarcomas [56], with notable enrichment of expression of vascular-specific receptor tyrosine kinases TIE1, KDR (VEGFR2), SNRK, TEK, and FLT1 (VEGFR1), and other genes that are prototypical endothelial markers including EPHA2 and PDGFβ. The overlap in the gene lists from Antonescu et al [56] and from our data supports the similarities between human angiosarcoma and canine hemangiosarcoma. However, an interesting contrast is Antonescu's observation that VEGF expression was lower in angiosarcoma than other soft tissue sarcomas, versus our observation showing enriched expression of VEGF in hemangiosarcoma cells as compared to non-malignant endothelial cells from splenic hematomas. This may be due simply to the relative comparisons of tumor vs. tumor (by Antonescu et al) and tumor vs. non-tumor (in this study), which also could explain the lack of enrichment for vascular-specific receptors in our study, as these molecules would be expressed both in hemangiosarcoma cells and in non-malignant endothelial cells. In this respect, an intriguing finding from our data was the specific enrichment of VEGFR1 in hemangiosarcoma cells derived from golden retriever tumors compared to hemangiosarcoma cells derived from tumors of dogs from other breeds [40], highlighting the potential utility of the organization and the relative homogeneity of dog breeds to understand how heritable factors might influence tumor pathogenesis.

We also were not surprised to find alterations in the expression of genes that contribute to malignant transformation and inflammation, including those that mediate cellular adhesion, stromal degradation or invasion (metalloproteinases), and the pathogenesis of leukemia [57-60]. For example, among the receptor tyrosine kinases found by Antonescu [56], TIE1 governs expression of inflammation-associated genes by endothelial cells [61]. IL8 and IL6, both of which were enriched in our hemangiosarcoma samples, are recurrently associated with inflammation that "benefits" tumors (Figure S3), and PTGS2 (a.k.a., COX-2) is the single most common tumor-associated pro-inflammatory mediator [16]. It is especially interesting that expression of PTGS2/COX-2 was enriched in our samples, as it was previously reported that the enzyme was undetectable by immunohistochemistry in formalin-fixed samples from canine hemangiosarcomas [62]. There are several non-mutually exclusive explanations for this finding, including greater sensitivity in the expression microarray platform than immunohistochemistry, inefficient translation or relatively short protein half-life for Cox-2 in these tumors, or induction of the gene when cells are removed from the tumor microenvironment. Additional work will be required to clarify the role of PTGS2/COX-2 in hemangiosarcoma.

As may be true for hypoxia response genes, upregulation of proinflammatory genes in hemangiosarcoma also could result from selective pressures to create a favorable microenvironment for growth and survival [63]. Tumors have been likened to "wounds that never heal" [64], which is reflected by shared expression of genes mediating breakdown of the extracellular matrix, productive chronic inflammation, and angiogenesis. For example FN-1, which was overexpressed in all hemangiosarcomas evaluated in this set of experiments, is involved in wound healing, blood coagulation, and cancer metastasis. FN-1 also increases MMP9 activity, which together with urokinase is involved in tumor cell invasion through the extracellular matrix [65,66]. The correlation between FN-1 and urokinase is interesting, since the survival rate of patients and dogs with angiosarcoma and hemangiosarcoma, respectively, is exceptionally poor due to its exceedingly high metastatic potential. The role of urokinase and FN-1 to promote the metastatic phenotype has been the subject of intense study in other tumors [46,65], but it remains to be examined in hemangiosarcoma.

On the other hand, angiogenic and inflammatory signatures might reflect the ontogeny of hemangiosarcoma, rather than selection in the tumor microenvironment. Inflammatory infiltrates are commonly seen in canine hemangiosarcoma, but rather than reflecting recruitment of tumor-associated macrophages and myeloid cells due to inflammation, perhaps leukocytes may actually be derived from a population of multipotent progenitor cells that give rise to hemangiosarcoma. Recent data suggest that classical cell markers for endothelial and myeloid origin cells are less tissue specific than historically thought [67], possibly due to a the existence of a shared hematopoietic/endothelial progenitor (the putative angioblast). We proposed recently that hemangiosarcomas might arise from such a cell [36], while Yoder et al described a similar population of myeloid cells that are intimate participants in blood vessel formation [37]. This cell is a "vascular mimic" that can express a variety of cell surface proteins associated with endothelial precursor cells (CD133, CD34, VEGFR2), but also proteins that belie hematopoietic origin (CD45, CD14, and CD115, the CSF1 receptor), that has phagocytic activity, and that does not contribute to the capillary endothelial layer in transplanted matrix. The enrichment of the adhesion molecule CD44, which in combination with PGE and Wnt-mediated signals may maintain slow cycling stem cell populations [68], support the possibility that tumor-initiating cells in hemangiosarcomas share properties that have been ascribed to "cancer stem cells" in other tumors.

We conclude that one single lineage may give rise to both endothelial and hematopoietic progenitors, or alternatively, that multiple lineages contribute to blood vessel formation, including one originating from a restricted angioblastic progenitor that gives rise to the endothelial lining cells and one originating from a myeloid progenitor that is responsible for creating (but not lining) vascular channels. In this latter scenario, plasticity of adult hematopoietic and mesenchymal stem cells would be limited, differentiation of myeloid progenitors into endothelial-like cells would have to reflect functional rather than ontogenetic plasticity, and we should consider the possibility that canine hemangiosarcoma, and by extension, human angiosarcoma, might represent a subtype of myeloid sarcomas. This interpretation is supported by the general enrichment of genes overexpressed in dendritic cells and in leukemia, as well as by enrichment of the patterning gene HOXA10 and by the specific downregulation of PLZF in hemangiosarcomas; both of which are involved in hematopoietic differentiation [45,69]. These results are especially significant in light of the poor response to treatments that presume canine hemangiosarcomas are tumors of blood vessels, and it may signal the need to revise the therapeutic approach to hemangiosarcoma and angiosarcoma as tumors of hematopoietic origin.

Preliminary data from our laboratories support the existence of rare progenitor cells in hemangiosarcoma that are responsible for propagating our cell lines *in vitro*. There also is evidence for cancer stem cells that are capable of differentiating along different developmental paths to give rise to endothelial cells in chronic myelogenous leukemia and Burkitt lymphoma [70,71]. Nevertheless, the possibility of vascular mimicry rather than true vascular differentiation cannot be excluded because the reverse outcome (endothelial tumors to hematopoietic cells) has not been documented.

To overcome the potential limitation from use of cell lines vs. intact tumors, we compared expression of a restricted, recurrently enriched signature from the hemangiosarcoma cell lines to whole tumor tissues. The data suggest that stromal and inflammatory cells can explain observed difference between these types of samples. Tumor cells modify the microenvironment and are themselves responsive to environmental cues. Nevertheless, to understand the contribution of the tumor cells to biological and pathological processes, it is important to examine the response in isolated cells. Microdissection of malignant cells from vascular tumors is difficult without retaining blood elements and normal angiogenic components that can be morphologically indistinguishable from the tumor cells. Conversely, cell lines provide a homogeneous, unlimited resource that can be extensively characterized with regard to ontogeny. The potential limitation of cell lines is further mitigated by the use of non-malignant controls to filter adaptation to *ex vivo *growth and by use of multiple samples. Finally, cell lines derived using our protocols retain the unique properties of the sample specimens and provide biologically relevant information.

## Conclusions

The data show that inflammation and angiogenesis are important processes in the pathogenesis of vascular tumors, but a definitive ontogeny of the cells that give rise to these tumors remains to be established. The data do not yet distinguish whether functional or ontogenetic plasticity creates this phenotype, although they suggest that cells which give rise to hemangiosarcoma modulate their microenvironment to promote tumor growth and survival. We propose that the frequent occurrence of canine hemangiosarcoma in defined dog breeds, as well as its similarity to homologous tumors in humans, offers unique models to solve the dilemma of stem cell plasticity and whether angiogenic endothelial cells and hematopoietic cells originate from a single cell or from distinct progenitor cells.

## Competing interests

The authors declare that they have no competing interests.

## Authors' contributions

BAT and JFM conceptualized the project and wrote the manuscript. BAT conceptualized performed, and analyzed the microarray experiments. TLP and SCT performed bioinformatic analyses. SPF designed and performed VHL cloning. MCS performed microarray experiments in tissues and IPA analysis. MMD was responsible for cell culture and for development and interpretation of immunoblots, and JWW validated or developed methods and completed immunostaining. JFM and LCS analyzed, scored, and interpreted immunostaining data. SRR designed experiments to define IL8 signaling networks. JES, DB, RMG, LEH, and JFM developed the experimental hypotheses. JES supervised Ras cloning experiments. LCS developed a conceptual translation of the data. GRC was responsible for statistical design and analysis. All authors read, edited, and approved the final manuscript. JFM verified the final content of the manuscript and bears responsibility for its accuracy.

## Pre-publication history

The pre-publication history for this paper can be accessed here:

http://www.biomedcentral.com/1471-2407/10/619/prepub

## Supplementary Material

Additional file 1 **Table S1 - Signalment (Demographics) of Dogs in Study**. Click here for file

Additional file 2 **Supplementary Methods (cloning canine VHL)**. Click here for file

Additional file 3 **Figure S1 - Sequence of canine VHL**. Click here for file

Additional file 4 **Figure S2 - Expression of Erk1/2 and pErk1/2 in canine hemangiosarcoma cells**. Click here for file

Additional file 5 **Table S2 - Genes expressed differentially between hemangiosarcoma tumors and non-malignant hematomas**. Click here for file

Additional file 6 **Table S3 - Functional Grouping of Genes Differentially Expressed in Hemangiosarcomas and Non-Malignant Splenic Hematomas**. Click here for file

Additional file 7 **Figure S3 - IL8 Signaling network in canine hemangiosarcoma**. Click here for file
