# Supplementary material for: Gene expression profiling identifies inflammation and angiogenesis as distinguishing features of canine hemangiosarcoma
Source: BMC Cancer. 2010 Nov 9;10:619. doi: 10.1186/1471-2407-10-619 (PMC2994824; doi:10.1186/1471-2407-10-619)
Supplement: Additional file 1 — Table S1 - Signalment (Demographics) of Dogs in Study. [file 1471-2407-10-619-S1.PDF]

Additional file 1 –Table S1 - Signalment (demographics) of dogs in study

Sample ID	Diagnosis	Breed	Sex	Age	Sample type
CHAD G4	Hemangiosarcoma	Golden retriever	Male	10	Cell line
CHAD G6	Hemangiosarcoma	Golden retriever	Female	12	Cell line
CHAD G8	Hemangiosarcoma	Golden retriever	Male	12	Cell line
FROG	Hemangiosarcoma	Golden retriever	Female	10	Cell line
JOURNEY	Hemangiosarcoma	Golden retriever	Female	11	Cell line
TUCKER	Hemangiosarcoma	Golden retriever	Male	6	Cell line
JOEY	Hemangiosarcoma	Rottweiler	Male	9	Cell line
DD-1	Hemangiosarcoma	Golden retriever X Great Pyrenees	Male	9	Cell line
CHAD P9	Hemangiosarcoma	Portuguese Water Dog	Male	9	Cell line
DAL-4	Hemangiosarcoma	Dalmatian	Male	7	Cell line
Pippin	Splenic hematoma	Golden retriever	Female	12	Cultured cells
Mariah*	Splenic hematoma	Labrador retriever	Female	12	Cultured cells
Baxter	Splenic hematoma	Poodle	Male	7	Cultured cells
Kylie	Splenic hematoma	Keeshond	Male	9	Cultured cells
OSCA-20	Osteosarcoma	Golden retriever	Male	7	Cell line
OSCA-23	Osteosarcoma	Golden retriever	Male	9	Cell line
OSCA-59	Osteosarcoma	Golden retriever	Female	9	Cell line
OSCA-75	Osteosarcoma	Golden retriever	Female	10	Cell line
OSCA-71	Osteosarcoma	Golden retriever	Female	7	Cell line
Rascal	ALL[a]	Golden retriever	Male	7	Primary cells
Maddie	CLL	Golden retriever	Female	9	Primary cells
Forrest	CLL	Golden retriever	Male	10	Primary cells
Benson	DLBCL	Golden retriever	Male	6	Primary cells
Cooper	DLBCL	Golden retriever	Male	6	Primary cells
Sally	DLBCL	Golden retriever	Female	10	Primary cells
Kaycee	DLBCL	Golden retriever	Female	14	Primary cells
Snuggles	DLBCL	Golden retriever	Male	14	Primary cells
Chieftain	DLBCL	Golden retriever	Male	10	Primary cells
Bitsy	MZL	Golden retriever	Female	10	Primary cells
Newman	TZL	Golden retriever	Male	10	Primary cells
Niki	TZL	Golden retriever	Female	10	Primary cells
Rose	TZL	Golden retriever	Female	10	Primary cells
Shilo	TZL	Golden retriever	Female	10	Primary cells
Thunder	TZL	Golden retriever	Male	10	Primary cells
Fire	TZL	Golden retriever	Male	11	Primary cells

*= not used is gene expression profiling experiments

[a]ALL = acute lymphoblastic leukemia, CLL = chronic lymphocytic leukemia, DLBCL = diffuse large B cell lymphoma, MZL = marginal zone lymphoma, TZL = T zone lymphoma
