# Supplementary material for: Gene expression profiling identifies inflammation and angiogenesis as distinguishing features of canine hemangiosarcoma
Source: BMC Cancer. 2010 Nov 9;10:619. doi: 10.1186/1471-2407-10-619 (PMC2994824; doi:10.1186/1471-2407-10-619)
Supplement: Additional file 2 — Supplementary Methods (cloning canine VHL). [file 1471-2407-10-619-S2.PDF]

Additional file 2 – Supplementary Methods

Cloning canine VHL. Based on the levels of VHL protein expression detected using immunohistochemistry (antibody G-7, Santa Cruz Biotechnology, Santa Cruz, CA, which is cross-reactive with pVHL from various species), we selected canine kidney as the most suitable sample to clone canine VHL. We used conserved sequences from aligned human, mouse, and rat VHL to design primer sets that would amplify 375 base pairs encompassing a partial cDNA sequence from mRNA (forward primer, position 424 in the human mRNA sequence: CCCTCCCAGGTCATCTTCTGC; reverse primer, position 799 in the human mRNA sequence: TCTGCACATTTGGGTGGTCTTCC) by RT-PCR, using an annealing temperature of 60°C. We verified the product using nested RT-PCR (annealing temperature 65°C) in a reaction to generate a 158 bp product (forward primer, position 550 in the human mRNA sequence: CGAGGTCACCTTTGGCTCTTCAG; reverse primer, position 708 in the human mRNA sequence: AACCTGGAGGCATCGCTCTTTC). The amplification products were sequenced as described [1]. The Genbank accession number for this partial VHL cDNA sequence is GU563722. We subsequently completed cloning the full-length gene, including identification of intron/exon boundaries from DNA by conventional PCR, by designing exon-specific primer sets based on comparative analyses with the human and murine sequences. The primers used to amplify exon 1, exon 2, and exon 3, respectively were forward primers (1) ATGCCCCGGAAGGCAGGGAGC, (2) GGTCACCTTTGGCTCTTCC, (3) GTGTATACTCTGAAAGAGCG, and reverse primers: (1) CTCGGTAGCTGTGGATGCGGC, (2) TGGCAGTGTGATGTTGGC, (3) TCAATTAAAATCCTCAGTC, respectively using annealing temperatures of 68°C, 65°C and 52°C. The Genbank accession number for the coding sequence in canine VHL exons 1, 2, and 3 is GU563723. The complete coding sequence of canine VHL (660 nucleotides from the ATG start codon to the TGA stop codon) was finally

confirmed by amplification of mRNA by RT-PCR using primers derived from the sequence of Kobayashi et al (Genbank accession AY764285). The forward primer, CGTTGTCTAGGCTCCGGG, started at position 19 in the Kobayashi sequence (23 bp upstream of the start site), and the reverse primer, GGCTGAGACTCAGGAGTGC, started at position 725 in the Kobayashi sequence (24 bp downstream of the stop codon). The annealing temperature used for this reaction was 60°C.

The predicted cDNA sequence from these reactions (see Additional File 3, Figure S1) aligned perfectly with the putative coding sequence for canine VHL in chromosome 20 from the canine genome assembly, as well as with the sequence submitted to Genbank by Kobayashi et al (accession AY764285). There was a single base pair substitution at position 603 (A → G) in our sequence (derived from tissues from 2 unrelated dogs) as compared to Kobayashi's sequence, leading to a single substitution in the translated amino acid sequence at position 202 (threonine → alanine). The predicted threonine at position 202 (our sequence) is conserved among human (accession NP_000542), chimp (accession XP_001144433), orangutan (accession NP_001126390), rhesus (accession XP_001090152), and cow (accession NP_001103489), with the rodent protein having a conserved substitution from threonine to serine. Thus, we believe the threonine residue at position 202 likely represents wild type canine VHL. The translated amino acid sequence for canine VHL is most similar to the human VHL isoform 1, with 95% identity in the conserved VHL domains (amino acids 58 - 211). In fact, conservation between human, rodent [2], and dog VHL between amino acid 62 and amino acid 195 was 99%, with a single conserved substitution (H→Y) at amino acid 125 (Figure S1). It is improbable (<5% chance) that this represents a polymorphic region, since the same sequence was obtained from 15 unrelated dogs.

References

1. Koenig A, Bianco SR, Fosmire S, Wojcieszyn J, Modiano JF: **Expression and significance of p53, Rb, p21/waf-1, p16/ink-4a, and PTEN tumor suppressors in canine melanoma**. *Vet Pathol* 2002, **39**(4):458-472.
2. Woodward ER, Buchberger A, Clifford SC, Hurst LD, Affara NA, Maher ER: **Comparative sequence analysis of the VHL tumor suppressor gene**. *Genomics* 2000, **65**(3):253-265.
