# Supplementary material for: Gene expression profiling identifies inflammation and angiogenesis as distinguishing features of canine hemangiosarcoma
Source: BMC Cancer. 2010 Nov 9;10:619. doi: 10.1186/1471-2407-10-619 (PMC2994824; doi:10.1186/1471-2407-10-619)
Supplement: Additional file 3 — Figure S1 - Sequence of canine VHL. [file 1471-2407-10-619-S3.PDF]

Additional file 3 - Figure S1

(A)

```
AY764285(Kobayashi)   1 CCCCGGCGGCGGGCCTCGCGTTGTCTAGGCTCCGGGAGGTAATGCCCCGG  50
GU563723              1                                          ATGCCCCGG   9

AY764285(Kobayashi)  51 AAGGCAGGGAGCGTGGAGGAGGCCGAGGCGGGCGCCGAGGAGGTGGGCGC 100
GU563723             10 AAGGCAGGGAGCGTGGAGGAGGCCGAGGCGGGCGCCGAGGAGGTGGGCGC  59

Kobayashi(AY764285) 101 CGAGGAGGTGGGCCCTGAAGAGTCCGGCGGGGAGGAGTCTGGCGCGGAGG 150
GU563723             60 CGAGGAGGTGGGCCCTGAAGAGTCCGGCGGGGAGGAGTCTGGCGCGGAGG 109

AY764285(Kobayashi) 151 AGTCCGGCCCGGAAGAGTCTGACCCGGAGGAGCCGGGCGCTGCGGCGGAG 200
GU563723            110 AGTCCGGCCCGGAAGAGTCTGACCCGGAGGAGCCGGGCGCTGCGGCGGAG 159

AY764285(Kobayashi) 201 ATGGAGGCCGGGCAGCCGCGGCCGGTGCTGCGCTCGGTGAACTCGTGCGA 250
GU563723            160 ATGGAGGCCGGGCAGCCGCGGCCGGTGCTGCGCTCGGTGAACTCGTGCGA 209

AY764285(Kobayashi) 251 GCCGTCCCAGGTCATCTTCTGCAACCGCAGCCCGCGCGTCGTGCTGCCGG 300
GU563723            210 GCCGTCCCAGGTCATCTTCTGCAACCGCAGCCCGCGCGTCGTGCTGCCGG 259

AY764285(Kobayashi) 301 TGTGGCTCAACTTCGACGGCGAGCCGCAGCCCTACCCGACGCTGCCGCCG 350
GU563723            260 TGTGGCTCAACTTCGACGGCGAGCCGCAGCCCTACCCGACGCTGCCGCCG 309

AY764285(Kobayashi) 351 GGCACGGGCCGCCGCATCCACAGCTACCGAGGTCACCTTTGGCTCTTCCG 400
GU563723            310 GGCACGGGCCGCCGCATCCACAGCTACCGAGGTCACCTTTGGCTCTTCCG 359

AY764285(Kobayashi) 401 AGATGCCGGGACATATGATGGGCTTCTGGTTAACCAAACTGAACTATTTG 450
GU563723            360 AGATGCCGGGACATATGATGGGCTTCTGGTTAACCAAACTGAACTATTTG 409

AY764285(Kobayashi) 451 TGCCATCTCTCAATGTTGATGGACAGCCAATTTTTGCCAACATCACACTG 500
GU563723            410 TGCCATCTCTCAATGTTGATGGACAGCCAATTTTTGCCAACATCACACTG 459

AY764285(Kobayashi) 501 CCAGTGTATACTCTGAAAGAGCGGTGCCTCCAGGTTGTCCGAAGCCTAGT 550
GU563723            460 CCAGTGTATACTCTGAAAGAGCGGTGCCTCCAGGTTGTCCGAAGCCTAGT 509

AY764285(Kobayashi) 551 CAAGCCTGAGAATTATAGGAGACTGGACATCGTGAGATCCCTCTATGAAG 600
GU563723            510 CAAGCCTGAGAATTATAGGAGACTGGACATCGTGAGATCCCTCTATGAAG 559

AY764285(Kobayashi) 601 ATCTGGAAGACCACCCGAATGTAAGGAAAGACCTGGAGCGGCTGGCACAG 650
GU563723            560 ATCTGGAAGACCACCCGAATGTAAGGAAAGACCTGGAGCGGCTGACACAG 609

AY764285(Kobayashi) 651 GAGCATATTGAAAATCAGCGAATGGAAGGGGAGACTGAGGATTTTAATTG 700
GU563723            610 GAGCATATTGAAAATCAGCGAATGGAAGGGGAGACTGAGGATTTTAATTG 659

AY764285(Kobayashi) 701 AAATTTGCACTCCTGAGTCTCAGCCTTTGATGGTACTGACTAATCTTGAT 750
GU563723            660 A                                                  660

AY764285(Kobayashi) 751 CTATATTTAGGACTGGTCACTTATTCTCACTTATTCTCACTTATTCAAGG 800

AY764285(Kobayashi) 801 TGTCTCATTCTCAGAGTAAAAAATCCTCCATTGCTTAAAGGAAAGTTAAC 850

AY764285(Kobayashi) 851 TGACTGCACTAGGCATTATGATGTTTCGGGGCAAATATCACAAGATGTAA 900

AY764285(Kobayashi) 901 CTTAATGCCTGCCCCTTCTAGAAGTATTCTTCAAATAGTT           940
```

(B)

```
AY764285(Kobayashi)    1 MPRKAGSVEEAEAGAEEVGAEEVGPEESGGEESGAEESGPEESDPEEPGA  50
GU563723               1 MPRKAGSVEEAEAGAEEVGAEEVGPEESGGEESGAEESGPEESDPEEPGA  50

AY764285(Kobayashi)   51 AAEMEAGQPRPVLRSVNSCEPSQVIFCNRSPRVVLPVWLNFDGEPQPYPT 100
GU563723              51 AAEMEAGQPRPVLRSVNSCEPSQVIFCNRSPRVVLPVWLNFDGEPQPYPT 100

AY764285(Kobayashi)  101 LPPGTGRRIHSYRGHLWLFRDAGTYDGLLVNQTELFVPSLNVDGQPIFAN 150
GU563723             101 LPPGTGRRIHSYRGHLWLFRDAGTYDGLLVNQTELFVPSLNVDGQPIFAN 150

AY764285(Kobayashi)  151 ITLPVYTLKERCLQVVRSLVKPENYRRLDIVRSLYEDLEDHPNVRKDLER 200
GU563723             151 ITLPVYTLKERCLQVVRSLVKPENYRRLDIVRSLYEDLEDHPNVRKDLER 200

AY764285(Kobayashi)  201 LAQEHIENQRMEGETEDFN 219
GU563723             201 LTQEHIENQRMEGETEDFN 219
```

Figure S1 - Sequence of canine VHL.

Panel (A) shows the nucleic acid sequence deposited in Genbank (ID AY764285) by Kobayashi et al ([1], Top), and the sequence we obtained, described in Materials and Methods (Genbank ID GU563723, Bottom). The single base pair difference between the sequences (A → G at position 603) is marked in red. Panel (B) shows the translated amino acid sequence for both sequences. The single amino acid substitution at position 202 (T → A) is marked in red.

Reference

1. **Canis familiaris von Hippel-Lindau disease tumor suppressor (VHL) mRNA, complete cds [http://www.ncbi.nlm.nih.gov:80/nuccore/AY764285.1?ordinalpos=1&itool=EntrezSystem2.PEntrez.Sequence.Sequence_ResultsPanel.Sequence_RVDocSum]**
