# Supplementary figures and images for: Gene expression profiling identifies inflammation and angiogenesis as distinguishing features of canine hemangiosarcoma
Source: BMC Cancer. 2010 Nov 9;10:619. doi: 10.1186/1471-2407-10-619 (PMC2994824; doi:10.1186/1471-2407-10-619)

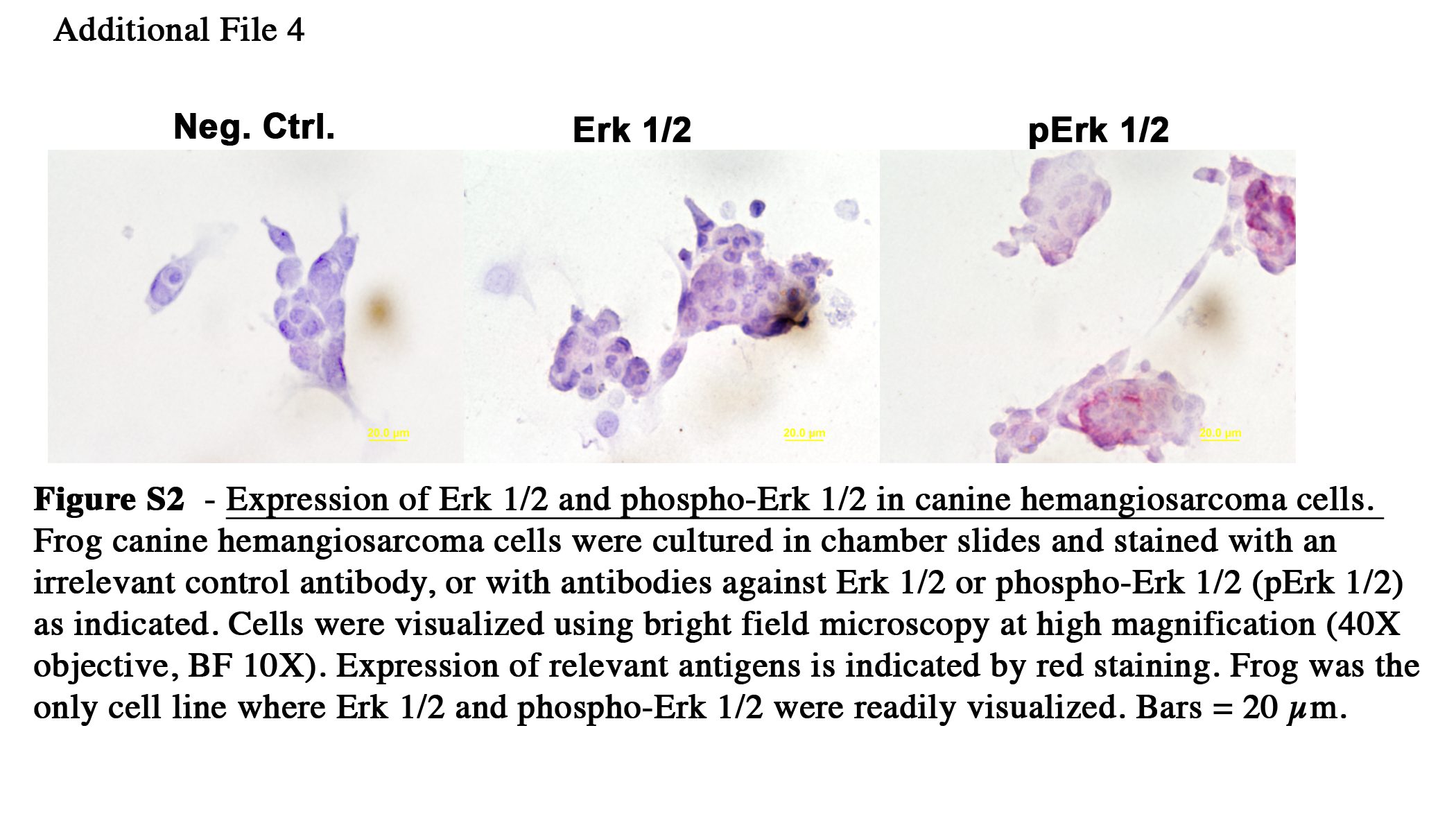

Supplement: Additional file 4 — Figure S2 - Expression of Erk1/2 and pErk1/2 in canine hemangiosarcoma cells. [file 1471-2407-10-619-S4.TIFF]

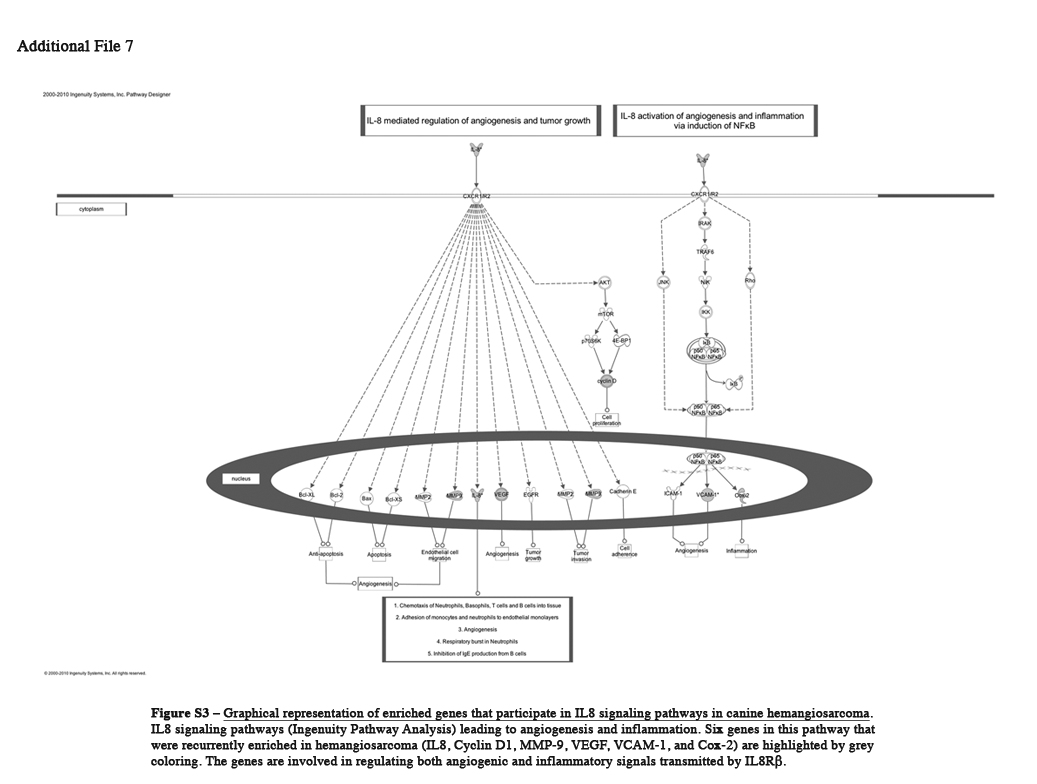

Supplement: Additional file 7 — Figure S3 - IL8 Signaling network in canine hemangiosarcoma. [file 1471-2407-10-619-S7.TIFF]
