# Supplementary material for: Gene expression profiling identifies inflammation and angiogenesis as distinguishing features of canine hemangiosarcoma
Source: BMC Cancer. 2010 Nov 9;10:619. doi: 10.1186/1471-2407-10-619 (PMC2994824; doi:10.1186/1471-2407-10-619)
Supplement: Additional file 5 — Table S2 - Genes expressed differentially between hemangiosarcoma tumors and non-malignant hematomas. [file 1471-2407-10-619-S5.PDF]

Additional File 5 - Table S2 - Genes expressed differentially between hemangiosarcoma tumors and non-malignant hematomas[a]

Number	Probe Set ID	Gene Title	Gene Symbol	p-value	Fold Change
1	Cfa.14762.1.S1_at	Calcineurin-like phosphoesterase domain containing 1	CPPED1	1.0E-04	-1.64
2	Cfa.15237.2.S1_x_at	Golgi integral membrane protein 4	GOLIM4	1.5E-04	-1.67
3	Cfa.15237.2.S1_a_at	Golgi integral membrane protein 4	GOLIM4	3.3E-04	-2.01
4	CfaAffx.22508.1.S1_s_at	Golgi integral membrane protein 4	GOLIM4	3.8E-04	-1.92
5	CfaAffx.24830.1.S1_at	Potassium voltage-gated channel, Isk-related family, member 4	KCNE4	7.9E-05	-2.42
6	CfaAffx.29263.1.S1_s_at	Zinc finger protein 185 (LIM domain)	ZNF185	3.3E-04	-2.91
7	Cfa.2324.1.A1_at	Transcribed locus	---	9.8E-07	-16.0
8	CfaAffx.30551.1.S1_at	WAP four-disulfide core domain 1	WFDC1	3.5E-06	-21.2
9	Cfa.11092.1.A1_at	Transcribed locus, strongly similar to NP_067020.2 WAP four-disulfide core domain	---	7.1E-06	-60.3
10	CfaAffx.19341.1.S1_at	Syntaxin binding protein 6 (amisyn)	STXBP6	2.0E-04	-2.33
11	CfaAffx.2623.1.S1_s_at	Similar to Protein C5orf5 (GAP-like protein N61)	LOC474690	1.2E-04	-1.93
12	Cfa.10703.1.A1_at	---	---	4.7E-05	-1.74
13	CfaAffx.6624.1.S1_s_at	Neuropilin 1	NRP1	1.1E-04	-3.61
14	Cfa.5536.1.A1_at	v-Myc myeloctomatosis viral related oncogene, neuroblastoma derived (avian)	MYCN	3.8E-04	-4.23
15	Cfa.10175.1.A1_at	Transcribed locus	---	3.8E-04	-5.90
16	CfaAffx.7397.1.S1_s_at	N-acetylated-alpha-linked acidic dipeptidase II	NAALAD2	1.5E-04	-2.07
17	Cfa.16449.1.A1_at	Transcribed locus, weakly similar to XP_531090.1	---	3.5E-07	-10.4

		PREDICTED: hypothetical protein			
18	CfaAffx.20922.1.S1_at	Zinc finger and BTB domain containing 16	ZBTB16	1.8E-04	-6.51
19	Cfa.2334.1.S1_at	Transcribed locus	---	3.1E-04	-1.69
20	CfaAffx.28472.1.S1_at	Receptor tyrosine kinase-like orphan receptor	ROR1	3.0E-04	-3.00
21	Cfa.16368.1.S1_at	Transcribed locus	---	3.7E-04	-8.35
22	CfaAffx.1441.1.S1_at	ADAM metallopeptidase with thrombospondin type 1 motif, 2	ADAMTS	1.6E-06	-5.27
23	Cfa.6326.1.A1_x_at	ADAM metallopeptidase with thrombospondin type 1 motif, 2	ADAMTS	2.2E-05	-4.43
24	Cfa.6326.1.A1_s_at	ADAM metallopeptidase with thrombospondin type 1 motif, 2	ADAMTS	2.9E-05	-8.07
25	CfaAffx.26569.1.S1_s_at	Myosin, heavy chain 10, non-muscle	MYH10	4.0E-05	-5.65
26	Cfa.6225.1.A1_at	Tumor necrosis factor receptor superfamily, member 14 (herpesvirus entry mediator)	TNFRSF14	3.6E-04	-3.12
27	CfaAffx.18191.1.S1_s_at	Myeloid cell nuclear differentiation antigen	MNDA	3.8E-04	-2.77
28	Cfa.9955.1.A1_at	---	---	1.4E-04	-2.39
29	CfaAffx.31100.1.S1_s_at	---	---	1.3E-04	-3.78
30	CfaAffx.13033.1.S1_s_at	Peptidase inhibitor 15	PI15	2.9E-04	-14.1
31	CfaAffx.13587.1.S1_at	ADAM metallopeptidase with thrombospondin type 1 motif, 5	ADAMTS5	8.8E-06	-5.37
32	Cfa.11011.1.A1_at	---	---	2.3E-04	-43.3
33	Cfa.151.1.S1_s_at	Monoamine oxidase A	MAOA	6.9E-05	-46.67
34	CfaAffx.22286.1.S1_at	Monoamine oxidase A	MAOA	2.1E-04	-60.6
35	Cfa.3909.1.S1_at	Platelet-derived growth factor receptor, beta polypeptide	PDGFRB	2.3E-04	-5.07
36	CfaAffx.27894.1.S1_s_at	Platelet-derived growth factor receptor, beta polypeptide	PDGFRB	2.4E-04	-2.38

37	CfaAffx.27700.1.S1_s_at	Cytospin B	CYTSB	2.8E-04	-2.25
38	CfaAffx.8555.1.S1_at	Hypothetical protein LOC611421	LOC611421	1.2E-04	-2.01
39	Cfa.8528.1.A1_at	Hypothetical protein LOC611704	LOC611704	1.4E-04	8.14
40	Cfa.2080.1.S1_at	Transcribed locus	-	3.3E-04	4.90
41	CfaAffx.28496.1.S1_at	Oncostatin M receptor	OSMR	6.7E-05	4.55
42	CfaAffx.9371.1.S1_s_at	Kinesin family member 5C	KIF5C	5.1E-05	3.90
43	Cfa.20479.1.S1_at	Kinesin family member 5C	KIF5C	9.8E-06	4.79
44	Cfa.10404.1.S1_at	Transcribed locus	---	1.5E-04	12.8
45	Cfa.363.3.S1_a_at	Similar to plasticity-related protein 3	LOC479934	2.2E-05	15.1
46	CfaAffx.30692.1.S1_s_at	Similar to plasticity-related protein 3	LOC479934	3.7E-05	17.6
47	Cfa.363.2.S1_at	Similar to plasticity-related protein 3	LOC479934	4.4E-05	13.6
48	CfaAffx.24616.1.S1_s_at	EPH receptor A2	EPHA2	1.9E-04	3.78
49	Cfa.2977.1.A1_s_at	ER degradation enhancer, mannosidase alpha-like 2	EDEM2	1.4E-04	1.71
50	Cfa.1749.1.A1_at	Transmembrane protein 41B	TMEM41B	3.6E-04	2.72
51	Cfa.3680.1.S1_s_at	TIMP metallopeptidase inhibitor 1	TIMP1	2.0E-04	4.88
52	Cfa.15207.2.S1_s_at	Transmembrane protein 41B	TMEM41B	2.5E-04	7.59
53	Cfa.3122.1.S1_at	Transcribed locus	---	1.4E-04	6.77
54	Cfa.1222.1.A1_at	Transcribed locus	-	8.0E-05	11.6
55	CfaAffx.1031.1.S1_at	Docking protein 6	DOK6	9.8E-05	9.88
56	Cfa.20638.1.S1_at	Carnitine palmitoyltransferase 1C	CPT1C	7.4E-05	2.83
57	Cfa.15616.1.A1_at	Transcribed locus	-	1.7E-04	2.23
58	Cfa.3800.2.S1_at	CD44 antigen	CD44	1.1E-04	2.25

[a]Gene list from Figure 3A identified by Affymetrix probe ID, gene title, and gene symbol. The p-values were calculated after verifying the data were normally distributed using Student's T-test; mean fold change reflects the average expression in hemangiosarcoma cells over the average expression in splenic hematoma cells
