# Supplementary material for: Gene expression profiling identifies inflammation and angiogenesis as distinguishing features of canine hemangiosarcoma
Source: BMC Cancer. 2010 Nov 9;10:619. doi: 10.1186/1471-2407-10-619 (PMC2994824; doi:10.1186/1471-2407-10-619)
Supplement: Additional file 6 — Table S3 - Functional Grouping of Genes Differentially Expressed in Hemangiosarcomas and Non-Malignant Splenic Hematomas. [file 1471-2407-10-619-S6.PDF]

Additional file 6 –Table S3 – Functional grouping of genes differentially expressed in hemangiosarcomas and non-malignant splenic hematomas[a]

Function	Gene title
Metabolic processing	Similar to ADAMTS-2 precursor
	Similar to ADAMTS-5 precursor
	Similar to Kinesin heavy chain isoform 5C
	Similar to carnitine palmitoyltransferase 1C
	Similar to potassium voltage-gated channel, Isk-related subfamily, gene 4
	Similar to ER degradation-enhancing-mannosidase-like protein 2
	Hypothetical protein LOC611704
	Similar to golgi phosphoprotein 4
	Similar to N-acetylated-alpha-linked acidic dipeptidase II (NAALADase II)
	Similar to syntaxin binding protein 6 (amisyn)
	TIMP metallopeptidase inhibitor 1
	Monoamine oxidase A
	Similar to transmembrane protein 41B
	Similar to Tyrosine-protein kinase transmembrane receptor ROR1 precursor
Cell-cell interaction	Similar to Kinesin heavy chain isoform 5C
	Similar to plasticity-related protein 3
	Similar to docking protein 5-like
	CD44 antigen
	Similar to Neuropilin-1 precursor (Vascular endothelial cell growth factor 165 receptor)
	Monoamine oxidase A
	Similar to transmembrane protein 41B
	Similar to spectrin domain with coiled-coils 1
	Similar to protease inhibitor 15 preproprotein
	Similar to myosin, heavy polypeptide 10, non-muscle
Survival/apoptosis	Similar to oncostatin M receptor
	Similar to Neuropilin-1 precursor (Vascular endothelial cell growth factor 165 receptor)
	Similar to ephrin receptor EphA2
	TIMP metallopeptidase inhibitor 1
	Platelet-derived growth factor receptor, beta polypeptide
	Similar to Tumor necrosis factor receptor superfamily member 14 precursor
Signaling/cell cycle	Similar to WAP four-disulfide core domain 1 precursor
	Transcribed locus, strongly similar to NP_067020.2 WAP four-disulfide core domain
	Similar to Kinesin heavy chain isoform 5C
	Similar to Neuropilin-1 precursor (Vascular endothelial cell growth factor 165 receptor)
	Similar to syntaxin binding protein 6 (amisyn)

	Platelet-derived growth factor receptor, beta polypeptide
	Similar to Zinc finger protein 185 (LIM-domain protein ZNF185) (P1-A)
	Similar to myosin, heavy polypeptide 10, non-muscle
Development	Similar to Neuropilin-1 precursor (Vascular endothelial cell growth factor 165 receptor)
	Similar to ephrin receptor EphA2
Angiogenesis	Similar to Neuropilin-1 precursor (Vascular endothelial cell growth factor 165 receptor)
	Similar to ephrin receptor EphA2
Transcription	Similar to ephrin receptor EphA2
	Similar to N-myc proto-oncogene protein
	Similar to myeloid cell nuclear differentiation antigen
Immune response	CD44 antigen
	Similar to Tumor necrosis factor receptor superfamily member 14 precursor

[a]Some genes are included in multiple functional categories
